# Fabrication of Stable Oleofoams with Sorbitan Ester Surfactants

**DOI:** 10.1021/acs.langmuir.2c02413

**Published:** 2022-11-21

**Authors:** Yu Liu, Bernard P. Binks

**Affiliations:** Department of Chemistry, University of Hull, Hull HU6 7RX, U.K.

## Abstract

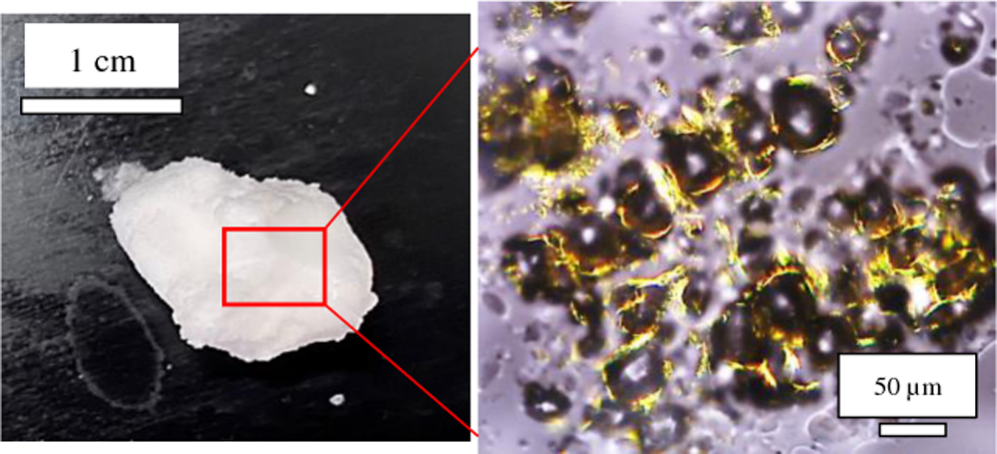

Sorbitan esters have been extensively used as surfactants to stabilize emulsions in many fields. However, the preparation of an oleofoam with sorbitan ester alone has not been reported. Here, we apply a novel protocol to fabricate stable oleofoams of high air volume fraction from mixtures of vegetable oil and sorbitan ester. To incorporate more air bubbles into the oil matrix, aeration is first carried out in the one-phase region at high temperatures, during which the highest over-run can reach 280%. Due to foam instability at high temperatures, the foam is then submitted to rapid cooling, followed by storage at low temperatures. For high-melting sorbitan monostearate, the resulting foams containing many crystal-encased air bubbles are ultrastable to drainage, coarsening, and coalescence for several months. On the contrary, the cooled foams with low-melting sorbitan monooleate go through a gradual decay lasting for more than 1 month. We highlight the importance of hydrogen bond formation between surfactant and oil in enhancing foam stability. The generic nature of the above findings is demonstrated by preparing oil foams with various vegetable oils and sorbitan monooleate.

## Introduction

Fats and oils are one of the most important macronutrients in the human diet, providing the highest fraction of total daily caloric intake.^1^ Of particular focus in the food industry is saturated fats since they are prevalently used as an alternative to trans fats.^2,3^ The use of saturated fats has two drawbacks, however: (i) their long-term intake may cause negative cardiovascular effects, and (ii) their excessive consumption may lead to obesity.^2,4^ Considering this, a dietary guideline from the Food and Agriculture Organization recommended that the daily consumption of saturated fats should be restricted to less than 10% relative to total calories.^5^ Thus, a great deal of effort has been devoted by food scientists to find ways to re-formulate food products with a better nutritional profile, *e.g.*, a lower level of saturated fats, while maintaining the desirable functionality endowed by saturated fats.

Oleogelation of edible oils using gelators of a natural or synthetic origin has been regarded as a promising oil-structuring technique due to its potential to reduce saturated fat content, improving nutritional and technological appeal, and imparting impressive rheological and sensorial properties.^2,6−11^ Commonly, oleogelation can convert ≥90 wt % of a flowable edible oil into an elastic gel with relatively low gelator concentration, during which a three-dimensional (3D) gelator network is formed due to noncovalent, *e.g.*, van der Waals, forces. Nevertheless, it still involves a large amount of edible oil during their formulation, the calorific content of which is similar to that of solid fat. Incorporating air bubbles into an oleogel matrix is a viable route to reduce its calorific content while maintaining or enhancing its rheological and mouthfeel properties.^12^ For example, aerated chocolate has a better mouthfeel and lower fat content than the nonaerated equivalent and patents have appeared.^13,14^ In these structured oil systems, air bubbles are stabilized by adsorbed lipid crystals akin to Pickering-like stabilization.^15^ In addition, excess lipid crystals present in the continuous phase can lead to the arrest of gravity-driven phase separation. Until now, the investigated lipids used to stabilize such oleofoams include monoglycerides (MAGs),^15−18^ diglycerides (DAGs),^19^ triglycerides (TAGs),^20−25^ fatty acids and fatty alcohols,^26−28^ and sucrose esters.^29^

Sorbitan esters (Spans) are biodegradable nonionic surfactants with sorbitol as the hydrophilic headgroup and fatty acid chains as the hydrophobic groups.^30^ They are widely used as emulsifying agents in the industries of food, cosmetics, and pharmaceuticals.^30−32^ The hydrophile–lipophile balance (HLB) number and the physicochemical properties of Spans vary, depending on the degree of esterification and nature of the alkyl chains. For example, Span 60 (sorbitan monostearate, HLB no = 4.7) is solid at room temperature due to its relatively long saturated fatty acid chain, while Span 80 (sorbitan monooleate, HLB no = 4.3) is liquid at ambient temperature because of its unsaturated fatty acid chain. In recent work on foaming of vegetable oils with sucrose ester surfactant, it was demonstrated that the carbonyl groups of TAG molecules form H-bonds with the hydroxyl groups of the surfactant.^29^ The molecular complexes can adsorb readily at the air–oil surface, rendering efficient foam formation upon whipping at high temperatures.^29^ Subsequent rapid cooling then induces crystal formation *in situ*, thereby endowing the foam with long-term stability. In comparison to previously published work on oleofoams,^15−28^ this foaming strategy has the advantage of achieving much higher over-run within a certain aeration time. The prerequisite is that the oil-soluble surfactant should contain hydroxyl groups. Moreover, the alkyl chain lengths of the TAG and surfactant should be comparable to promote interfacial crystallization on cooling.^33^ Based on this, we realized that the sorbitol headgroup of sorbitan monoester is rich in hydroxyl groups. In addition, the fatty acid chain length of common sorbitan monoesters, *e.g.,* Span 60, C18, is comparable to that of chains in typical vegetable oils, *e.g.*, sunflower oil. Can stable oil foams of high air volume fraction be obtained from Span surfactant + liquid vegetable oil mixtures?

Here, we select two typical sorbitan monoester surfactants, Span 60 and Span 80, which have been widely used as emulsifiers to stabilize water-in-oil emulsions.^31^ We first investigate the gelling and interfacial characteristics of Span 60 in rapeseed oil. Second, the effect of surfactant concentration and aeration temperature on the foaming behavior is studied. To yield stable oil foams, foams prepared at high temperatures are subjected to rapid quenching, followed by storing at low temperatures. Different techniques are used to unravel the underlying mechanisms, including Fourier transform infrared spectroscopy (FTIR), surface tension, rheology, differential scanning calorimetry (DSC), and polarized light microscopy. Finally, the foaming potential of mixtures of Span 80 and a variety of vegetable oils has been explored.

## Experimental Section

### Materials

The nonionic sorbitan esters, sorbitan monooleate (Span 80 HP-LQ-(MH), 0001684768) and sorbitan monostearate (Span 60 HP-PA-(MH), 0001314910), were from Croda, U.K., and Ireland. Span 80 is a viscous, yellow liquid, and Span 60 is a white solid at room temperature with a melting point of approx. 56–57 °C. The surfactants were used as received. Their chemical structures are given in Figure S1. Each surfactant molecule has three free hydroxyl groups. Rapeseed oil (SPLI-72119) was from Cargill NV, Belgium. It remains liquid down to at least −10 °C. The other vegetable oils are high oleic sunflower oil (002/12, Eulip), refined peanut oil (010/09, Eulip), extra virgin olive oil (SPLI-72120), and soybean oil (MKBP1045V, Sigma-Aldrich). Sesame oil and corn oil were purchased from a local Tesco store. The selected oils remain liquid down to at least 5 °C, and the triglyceride chains are abundant in long-chain unsaturated fatty acids, *i.e.*, oleic (C18:1) and linoleic (C18:2). All oils were passed once through an alumina column (Merck kGaA, 0.063–0.200 mm) to purify before use.

### Methods

#### Solubility of Span 60 in Rapeseed Oil

A mixture of Span 60 and rapeseed oil in a glass vial with a screw cap was placed in the well of a Grant R1 thermostat. The surfactant–oil mixture was first maintained at 80 ± 1 °C for 10 min with magnetic stirring (150 rpm), rendering a clear, homogeneous solution. It was then cooled gradually to target temperatures at 1 °C min^–1^ until reaching 20 °C. After aging at this temperature overnight (∼12 h), the sample was heated at 1 °C min^–1^ until it turned clear again.

#### Optical Microscopy

Optical microscopy was performed using a Leica DME optical microscope mounted with a Leica MC190 HD camera. Images were acquired by Leica Application Suite 4.12.0 software with and without polarizers. A drop of either surfactant in oil or oil foam (∼5 μL) was transferred by a micropipette onto the middle of a glass slide (76 mm × 26 mm) with a single circular cavity (15 mm) and then covered gently with a thin glass coverslip. The glass slide containing the sample was placed in the middle of a hot stage (Linkam PE120) connected to a digital controller (Linkam T95PE). All images were analyzed with ImageJ software (1.47 V) calibrated with a Pyser-SGI Graticule Limited. The number average bubble diameter of a foam sample was calculated from at least 200 representative bubbles.

#### Differential Scanning Calorimetry (DSC)

The crystallization and melting behavior of various samples, including neat Span 60 and Span 60 oleogels, was investigated using a PerkinElmer differential scanning calorimeter (DSC 4000) equipped with Pyris series software. The instrument was calibrated with indium, and nitrogen was used as the purge gas. The samples were weighed in aluminum pans using an analytical balance and then hermetically sealed at room temperature. An empty, sealed aluminum pan was used as a reference. All samples were heated from 20 to 80 °C and kept isothermally for 5 min at 80 °C prior to cooling to 20 °C. The temperature change rates for neat Span 60 and Span 60 oleogels were 5 and 3 °C min^–1^, respectively.

#### Rheology

The oscillatory rheology behavior of oleogels and oleofoams containing Span 60 was determined using a Bohlin rheometer (CVO120 High Resolution) with Bohlin software (R6.51.0.3). The serrated upper plate used was 40 mm in diameter, and the distance between the lower and upper plates was 1 mm. The temperature of the lower plate (connected to a water bath) was controlled by a Peltier system. For a temperature sweep, the program was as follows: (i) maintain at 80 °C for 5 min, (ii) cool from 80 to 7 °C at 1 °C min^–1^, (iii) equilibrate at 7 °C overnight, and (iv) warm from 7 to 80 °C at 1 °C min^–1^. The oscillation frequency *f* and oscillation stress τ were fixed at 1 Hz and 1 Pa, respectively. A stress sweep was done from 0.1 to 100 Pa at a fixed *f* of 1 Hz; a frequency sweep was carried out from 0. 1 to 100 Hz within the linear viscoelastic region (LVR).

#### Surface Tension

Air–oil surface tensions at different temperatures were measured using a Krüss K11 tensiometer and the Wilhelmy plate method. An oil solution/dispersion (∼10 mL) in a Petri dish was transferred to the well of a silicone oil jacket in the tensiometer at the target temperature. Before each measurement, the Pt plate was rinsed with ethanol and heated to glowing in a blue Bunsen flame.

#### Fourier Transform Infrared Spectroscopy (FTIR)

FTIR experiments were carried out using a Nicolet iS-5 FTIR spectrometer equipped with Omnic software. Neat Span 60 was loaded into the sampling area of a crystal plate using a MIRacle high-pressure clamp. It was then measured in the attenuated total reflectance (ATR) mode equipped with a MIRacle ATR accessory at room temperature. An oil solution (∼1 mL) sealed in a quartz cuvette (connected to a water bath) was fixed carefully in the path of the beam from the spectrometer under the absorption/transmittance mode. All measurements were performed with 32 scans, and background spectra were subtracted prior to measurements.

#### Foams Prepared and Stored at the Same Temperature

For Span 60, a glass beaker (250 mL) containing 30 g of its mixture with rapeseed oil was initially maintained at 80 °C under magnetic stirring (50 rpm), yielding a homogeneous, clear solution. The oil solution was then cooled to the target temperature in a water bath at 1 °C min^–1^. Likewise, a mixture of Span 80 and oil (30 g) in a glass beaker was submitted to magnetic stirring (50 rpm) overnight at room temperature. Both mixtures were then whipped with a handheld Vonshef single-beater electric whisk at 880 ± 88 rpm for 10 min. The over-run value of whipped oil was defined elsewhere.^29^ The fresh foams were then stored at the respective aeration temperature. To quantify the foam stability, the foam half-life and time for complete foam collapse were determined.

#### Fabrication of Stable Foams

Fresh Span 60 foams after 10 min of whipping at 80 °C were cooled rapidly in an ice bath at −5 °C (∼6 °C min^–1^), followed by storing in a fridge (internal temperature of 7 °C) or at room temperature (20 ± 2 °C). By contrast, freshly prepared foams with Span 80 at room temperature were cooled to −5 °C (*i.e.*, an intermediate temperature between the melting temperatures of neat Span 80 and rapeseed oil) in an ice bath of −5 °C. To monitor foam stability, the volume of foam, volume of drained oil, and average bubble size were determined as a function of storage time.

## Results and Discussion

We first explore the crystallization and melting behavior of Span 60 in rapeseed oil. Second, the effect of aeration temperature and surfactant concentration on the foaming behavior was investigated. To yield very stable foams, foams prepared at high temperatures are submitted to rapid cooling, followed by storing at low temperatures. Finally, the foaming potential of mixtures of Span 80 and a range of vegetable oils is described.

### Crystallization and Melting of Span 60 in Rapeseed Oil

#### Solubility and Crystal Morphology

**Figure 1:**
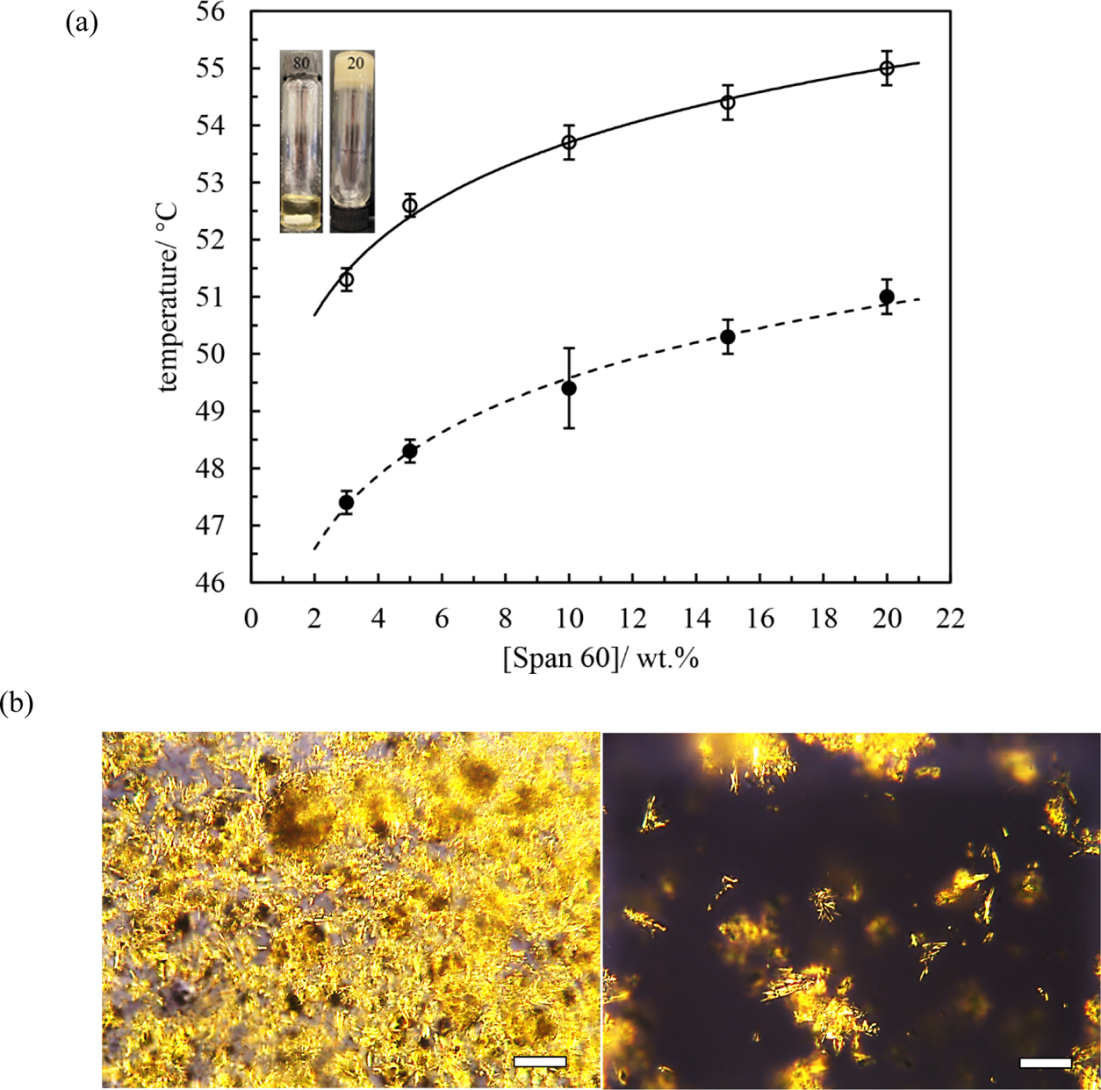
(a) Solubility diagram of Span 60 in rapeseed oil based on visual observations upon (●) cooling and (○) warming at rates of 1 °C min^–1^. Inset: the appearance of vials containing 20 wt % Span 60 in rapeseed oil at (left) 80 °C and (right) 20 °C. (b) Polarized light micrographs of rapeseed oil gel containing 20 wt % Span 60 at 20 °C, scale bars = 50 μm; (left) undiluted, (right) diluted.

Span 60 was first dissolved in rapeseed oil at 80 °C, followed by gradual cooling and subsequent warming at 1 °C min^–1^ (Figure 1a). During cooling, the solutions turned cloudy due to the formation of surfactant crystals in oil.^34−36^ After resting at room temperature overnight, the samples remained fluid for concentrations below 15 wt %, while oleogels that did not flow under gravity were obtained for concentrations ≥15 wt %. The concentration of 15 wt % of Span 60 was regarded as the critical gelling concentration (CGC) for rapeseed oil, similar to that for mustard oil (17 wt %),^34^ sesame oil (15 wt %),^35^ and olive oil (19 wt %).^36^ During warming, the samples became less turbid and viscous as crystals melted until transforming into clear oil solutions. The phase boundary separates a molecular solution of surfactant in oil at high temperatures from a dispersion of surfactant crystals in oil containing molecular surfactant at its solubility limit at low temperatures. Photos of rapeseed oil solution at 80 °C and rapeseed oil gel at 20 °C containing 20 wt % Span 60 are given as insets in Figure 1a. The temperature at which the mixture first became cloudy on cooling and the highest temperature where the cloudiness remained upon warming were recorded for different concentrations of Span 60 in rapeseed oil. These transition temperatures increase with Span 60 concentration. Thermal hysteresis can be observed between cooling and warming, in line with previous studies.^26,37^Figure 1b presents the polarized light micrographs of rapeseed oil gel containing 20 wt % Span 60 at 20 °C. For the undiluted sample, a dense network of birefringent Span 60 crystals is distributed randomly throughout the oil matrix. When the gel was diluted with oil, fiber-like crystals can be seen reminiscent of those observed for mustard oil gel of Span 60.^34^ The size of a single crystal is ≤50 μm.

#### Thermal Properties

**Figure 2:**
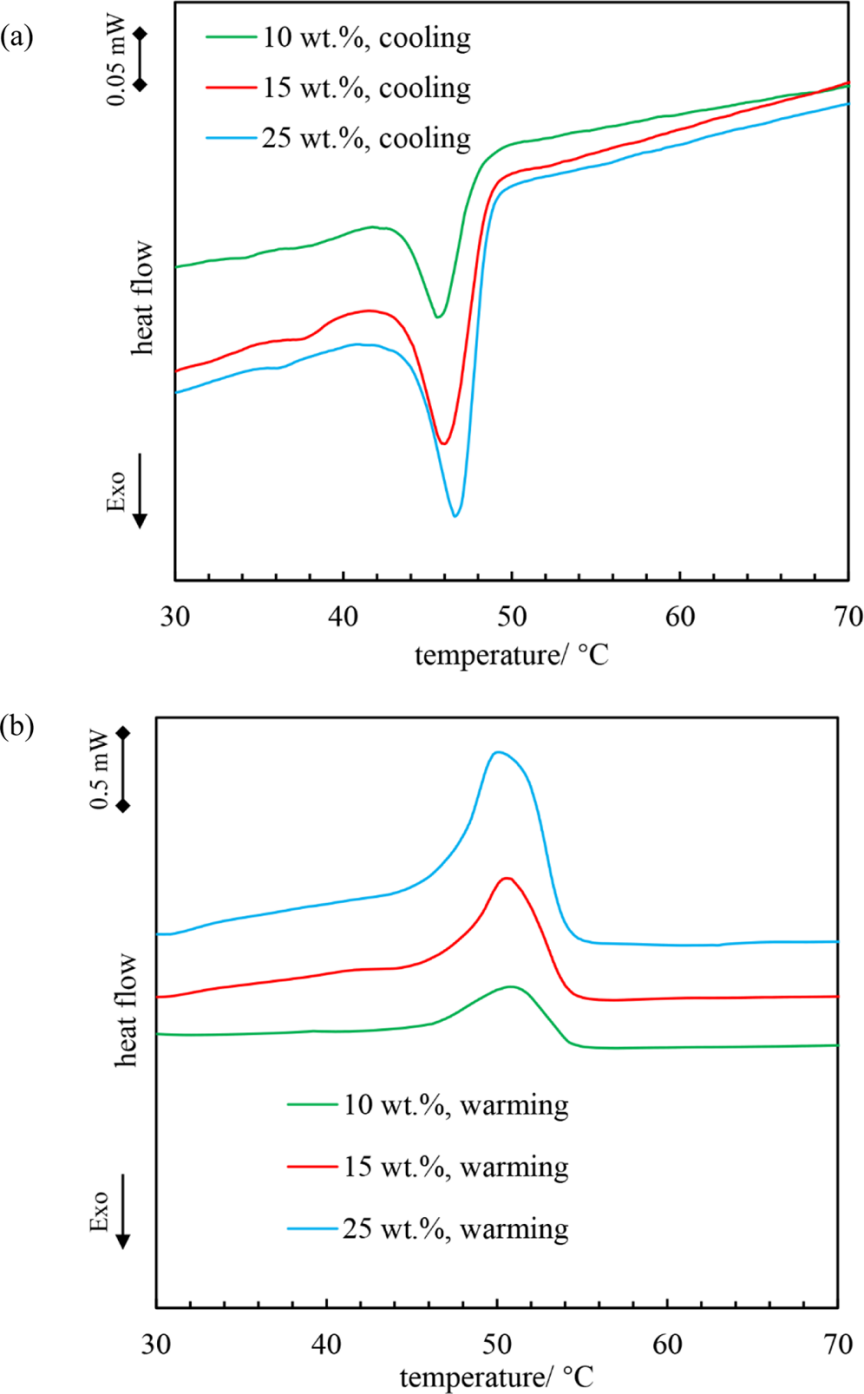
(a) Cooling and (b) warming thermograms of rapeseed oil gels containing different concentrations of Span 60 stored at ambient temperature (20 ± 2 °C). Temperature change rate was 3 °C min^–1^.

The thermal properties of neat Span 60 were investigated by DSC (Figure S2 and Table S1). During warming, the thermogram exhibits one distinct endothermic peak at 53.4 °C. Subsequent cooling shows two exothermic peaks at 48.2 and 40.7 °C. Similarly, Singh et al.^35^ reported an endothermic peak and an exothermic peak at 57.2 and 50.1 °C, respectively, during the heating and cooling cycles. The thermograms of oleogels containing different concentrations of Span 60 in rapeseed oil are given in Figures 2 and S3, and parameters derived from them are given in Table S2. The peak temperatures of melting and crystallization increase linearly with Span 60 concentration.^37^ Meanwhile, the enthalpy changes associated with melting and crystallization increase more or less linearly, suggesting the thermal stability of the oleogels is improved upon increasing the surfactant concentration.^34^

#### Rheology

**Figure 3:**
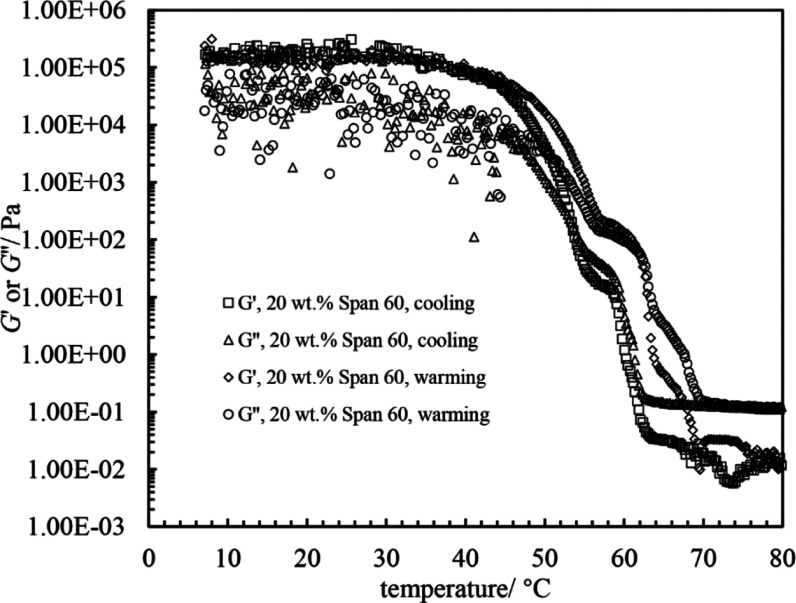
*G*′ and *G*″ *vs* temperature for 20 wt % Span 60 in rapeseed oil upon cooling and subsequent warming at 1 °C min^–1^. Measurements were done at a fixed stress τ = 1 Pa and a fixed frequency *f* = 1 Hz.

The rheological properties of oleogels containing different concentrations of Span 60 were investigated as a function of temperature. Oleogels stored at room temperature were heated to 80 °C, followed by cooling to 7 °C at 1 °C min^–1^, and then heated to 80 °C at 1 °C min^–1^ with *f* = 1 Hz and τ = 1 Pa. In Figure 3, we present the rheological data for a typical concentration above the CGC (*i.e.*, 20 wt %). During cooling, the initial values of *G*′ (elastic modulus) and *G*″ (viscous modulus) are almost independent of temperature and *G*′ < *G*″ corresponding to a weak liquid (<1 Pa). Upon cooling further, both values increase abruptly such that *G*′ > *G*″, corresponding to an elastic gel. For temperatures < 30 °C, *G*′ almost levels off at around 2 × 10^5^ Pa, characteristic of a firm gel. This trend is similar to that for monoglyceride–olive oil mixtures.^38^ During heating, *G*′ remains almost constant at temperatures < 30 °C with *G*′ > *G*″. Subsequently, both parameters display a slight decline followed by a significant decrease. For temperatures > 70 °C, *G*′ and *G*″ remain virtually constant with *G*′ < *G*″.

#### Surface Tension and FTIR Studies

**Figure 4:**
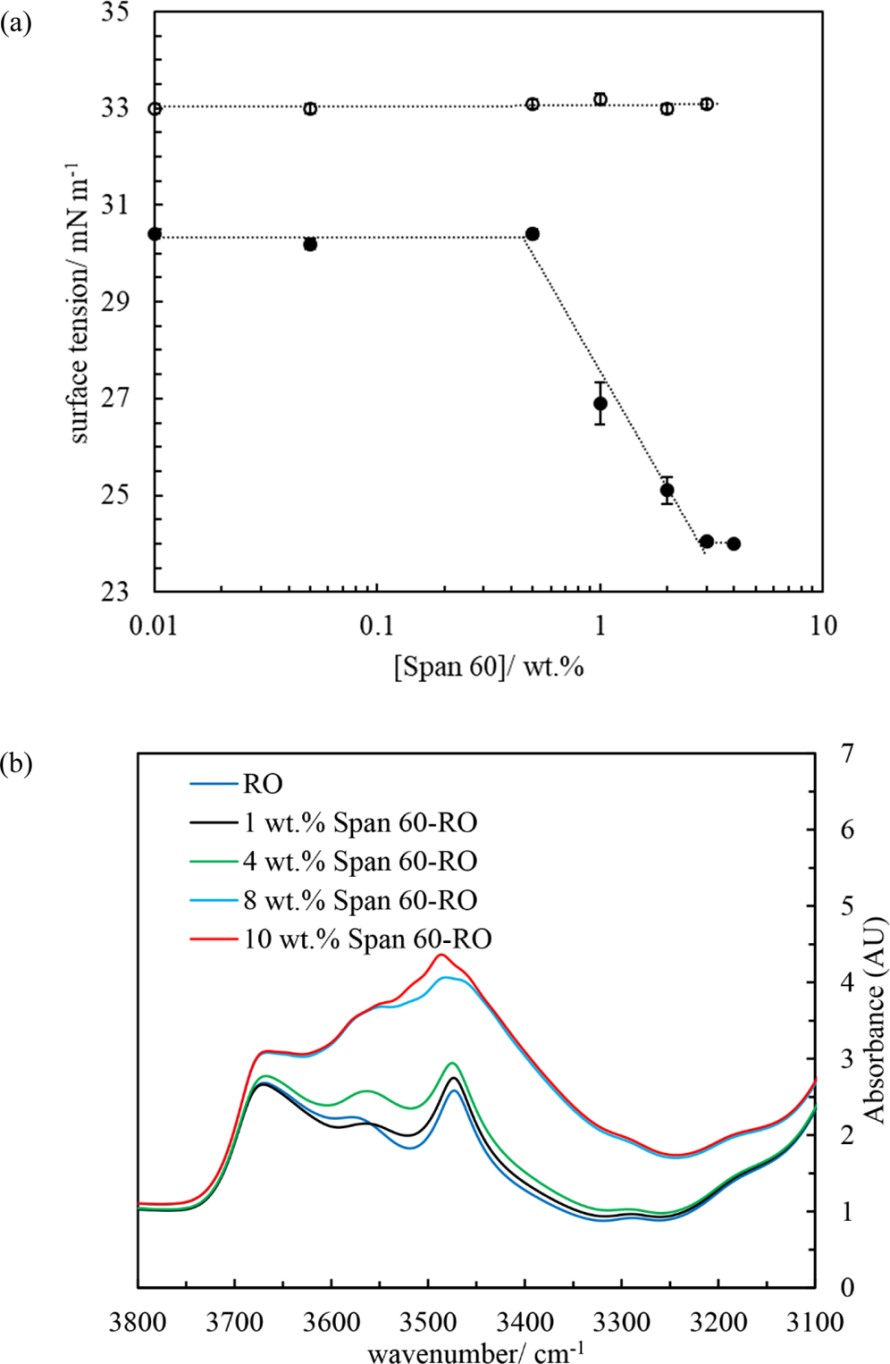
(a) Air–oil surface tension of Span 60 rapeseed oil mixtures at (○) 20 °C (two-phase region) and (●) 70 °C (one-phase region). Surface tension of neat rapeseed oil at 20 and 70 °C is 33.0 and 30.6 mN m^–1^, respectively. (b) FTIR spectra of rapeseed oil (RO) and rapeseed oil solutions of Span 60 at 70 °C.

Figure 4a gives the air–oil surface tension as a function of surfactant concentration at both 70 (one-phase) and 20 °C (two-phase). The surface tensions of neat rapeseed oil at 70 and 20 °C are 30.6 and 33.0 mN m^–1^, respectively. The latter values are similar to those from Xu et al.^39^ At 70 °C, the surface tension remains almost constant until 0.5 wt %. Above 0.5 wt %, the value decreases gradually, with Span 60 concentration reaching a constant value of ∼24 mN m^–1^ at and above 3 wt %. This critical surfactant concentration is comparable to that for corn oil solutions of Span 80 at 20 °C (3–4 wt %).^39^ This lowering of surface tension indicates that Span 60 molecules are surface-active at the air–oil surface.^39^ By contrast, at 20 °C, the surface tensions of oil dispersions of crystals are the same as that of neat oil, consistent with the fact that colloidal particles of size above tens of nanometers do not lower the surface tension.

The representative functional groups of and molecular interactions within neat Span 60, neat rapeseed oil, and rapeseed oil solutions of Span 60 were determined by FTIR spectroscopy. Figure S4a presents the FTIR spectrum of neat Span 60 at room temperature. It exhibits an −OH stretching peak at 3379 cm^–1^, and the C=O stretching peak can be observed at 1736 cm^–1^. Similar peaks (*i.e.*, ∼3400 and ∼1738 cm^–1^) were identified for neat Span 60 by Fathalla et al.^40^Figure 4b shows the FTIR absorbance spectra of rapeseed oil and rapeseed oil solutions of Span 60 at 70 °C in the one-phase region. Neat oil displays a minor peak at 3475 cm^–1^; this is due to the intermolecular H-bonds formed between the carbonyl groups in TAG and the hydroxyl groups of trace impurities, *e.g.*, fatty acid.^41^ For oil solutions of Span 60, the intensity and broadness of the peaks centered around 3475 cm^–1^ increase with surfactant concentration, arising from the intermolecular H-bonds formed between carbonyl groups of TAG molecules and hydroxyl groups of Span 60 molecules, *i.e.*, solvent–solute interactions.^42^Figure S4b shows that the absorbance of Span 60 rapeseed oil mixtures at 3475 cm^–1^ increases with surfactant concentration at 70 °C.

### Whipping Mixtures of Span 60 and Rapeseed Oil

#### Foams Prepared and Stored at the Same Temperature

**Figure 5:**
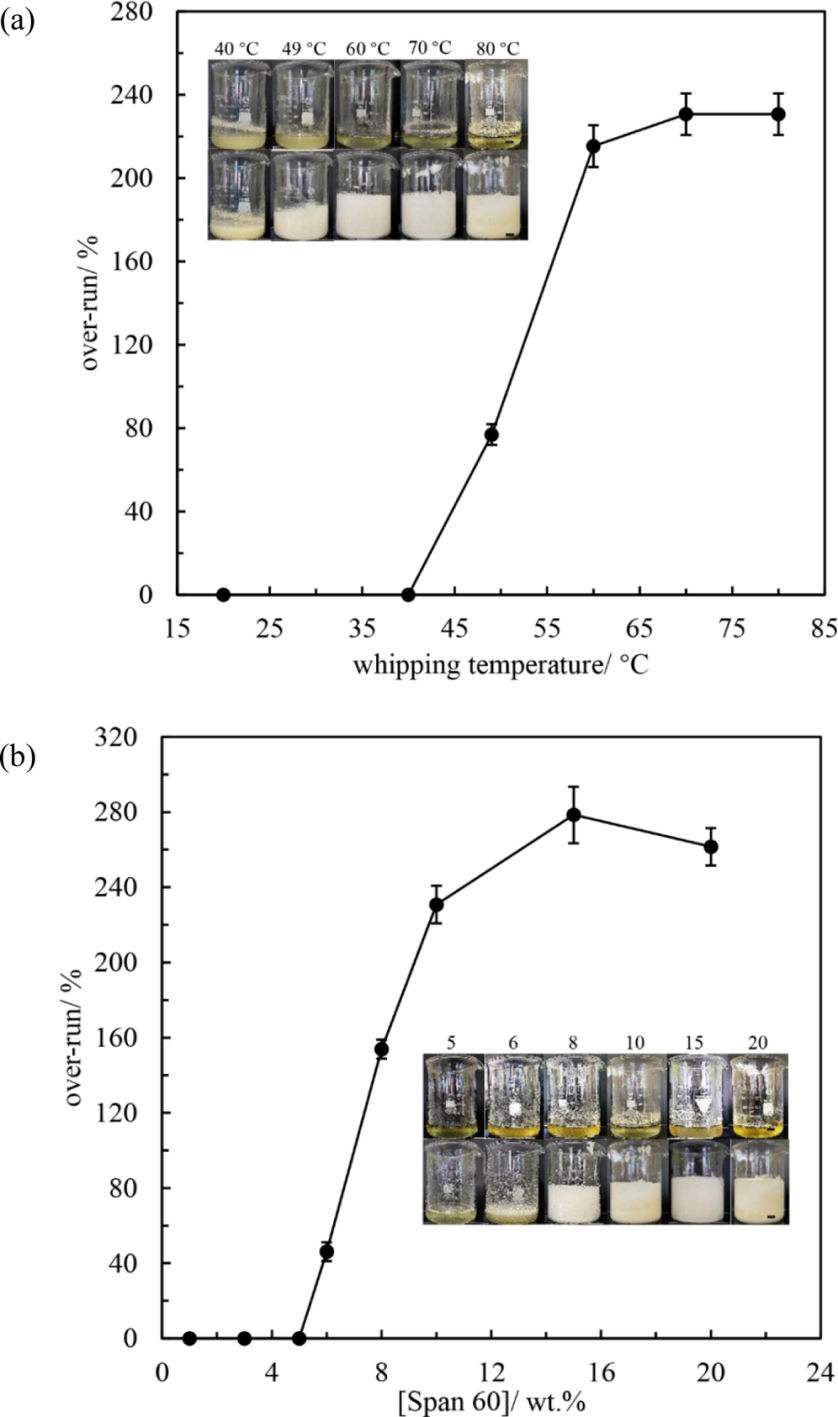
(a) Over-run *vs* whipping temperature for rapeseed oil containing 10 wt % Span 60 cooled from 80 °C and (b) over-run *vs* surfactant concentration in rapeseed oil at 80 °C. Insets: photos of surfactant–oil mixtures (upper) before and (lower) immediately after 10 min whipping; scale bars = 1 cm.

The foaming behavior of mixtures of Span 60 and rapeseed oil was explored as a function of aeration temperature and surfactant concentration. To investigate the effect of aeration temperature, the surfactant concentration was fixed at 10 wt %, which is above the critical surfactant concentration of 3 wt % determined from Figure 4a at 70 °C. The inset photos in Figure 5a present the appearance of rapeseed oil containing 10 wt % Span 60 before and immediately after 10 min whipping at some selected temperatures cooled from 80 °C. The samples at 60, 70, and 80 °C before whipping are clear solutions (one-phase), while those at 40 and 49 °C are cloudy dispersions (two-phase). No foam can be obtained at 40 °C. At temperatures above 40 °C, the foam volume increases and then almost levels off (Figure 5a). The effect of surfactant concentration was studied at a fixed temperature of 80 °C in the one-phase region. From the inset photos in Figure 5b, the minimum Span 60 concentration required to obtain oil foams is 6 wt %, which is higher than that using sucrose ester as surfactant (1–3 wt %).^29^ This is due to the fact that more free hydroxyl groups are available in each sucrose monoester molecule to form H-bonds with carbonyl groups of triglyceride molecules in vegetable oil. Increasing the surfactant concentration leads to an increase in the foam volume to a certain level. Overall, substantial foaming can be achieved in the one-phase region above a certain surfactant concentration, whereas there is little or no foaming in the two-phase region containing surfactant crystals. This foaming behavior is similar to that of sucrose ester in vegetable oil^29^ but contrasts that in many other systems where foaming only occurs in the presence of crystals. The highest over-run of whipped rapeseed oil is an impressive 278%, resulting in the highest volume fraction of incorporated air of 0.74.

**Figure 6:**
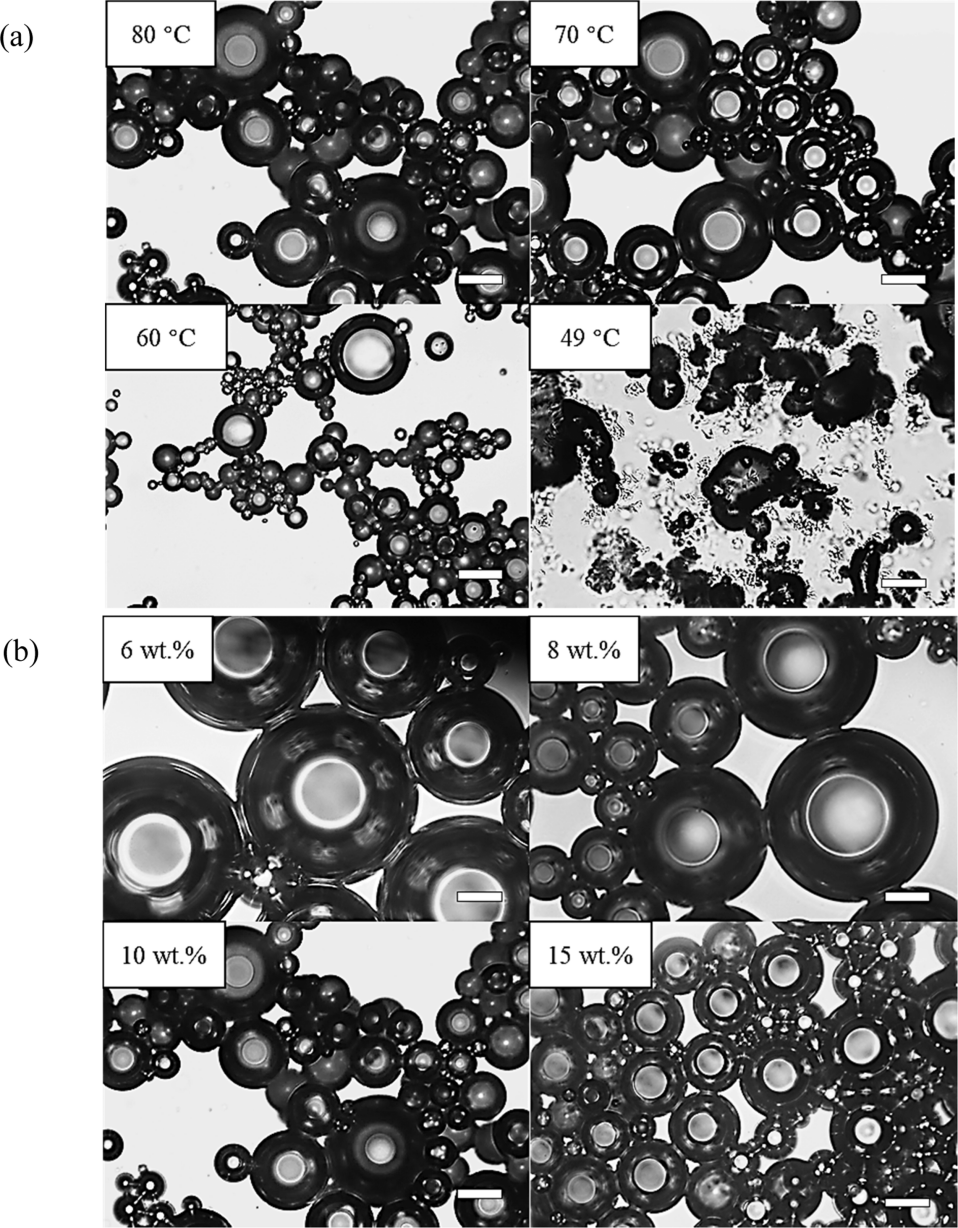
Optical micrographs of fresh rapeseed oil foams containing (a) 10 wt % Span 60 prepared at different temperatures and (b) different concentrations of Span 60 prepared at 80 °C. Scale bars = 50 μm.

Foams were stored at the respective aeration temperature. The foam half-life and time for complete foam collapse are shown as a function of storage temperature and surfactant concentration in Figure S5a,b, respectively. Both foam stability parameters increase up to a plateau value upon increasing either the storage (aeration) temperature or surfactant concentration. However, all of the foams are ultimately unstable, with no foam surviving after 3 days. Optical micrographs of some fresh oil foams are displayed in Figure 6. All foam bubbles are spherical with smooth surfaces except those prepared at 49 °C, which are nonspherical and possess textured surfaces arising from the presence of adsorbed surfactant crystals. The average bubble diameter decreases upon decreasing the whipping temperature or increasing the surfactant concentration. For example, the bubble diameter falls dramatically from ∼150 μm at 6 wt % to ∼30 μm at 15 wt % as more surfactant is available for stabilization.

#### Fabrication of Stable Foams

In recent work,^29^ a novel strategy was developed to prepare very stable oleofoams of high air volume fraction stabilized by sucrose ester. Foams prepared at high temperatures in the one-phase region were submitted to rapid cooling, followed by storing at low temperatures. The resulting foams were devoid of drainage, coarsening, or coalescence for several months. Crystallization of surfactant *in situ* around bubble surfaces produced crystal-stabilized foams. Taking advantage of the method described above, we chose to fabricate oil foams of Span 60 through 10 min whipping at 80 °C. The fresh foams were then quenched rapidly (∼6 °C min^–1^) in an ice bath of −5 °C and kept at either 7 °C or room temperature (20 °C).

**Figure 7:**
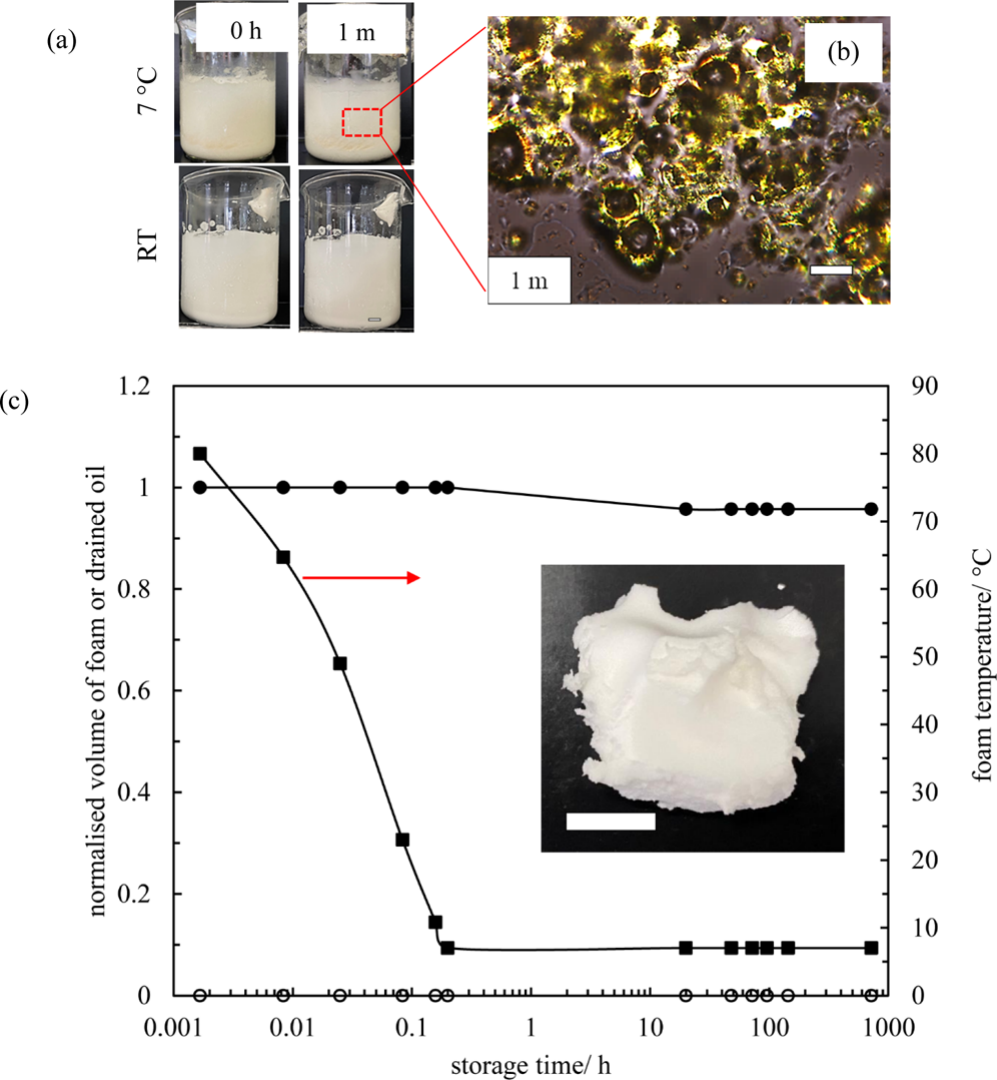
(a) Appearance of rapeseed oil foams containing 20 wt % Span 60 submitted to rapid cooling immediately after 10 min whipping. Foam prepared at 80 °C was cooled in an ice bath of −5 °C, followed by storing at (upper) 7 °C or (lower) room temperature (RT). Scale bar = 1 cm. (b) Polarized microscopy image of rapeseed oil foam for the system in panel (a) after 1-month storage at 7 °C. Scale bar = 50 μm. (c) Normalized volume of foam (●) and drained oil (○) and foam temperature (■) as a function of aging time for foam at 7 °C. Inset: photo of the foam after 3 months of storage; scale bar = 1 cm.

Figures S6 and S7 illustrate the appearance of rapeseed oil foams containing either 10 or 15 wt % Span 60 during storage at 7 °C, respectively. Drained oil can be observed at the vessel bottom on long-term storage. Meanwhile, the foam network is gradually fractured, possibly due to its intrinsic fragility.^29^ From the nonpolarized and polarized light micrographs, many nonspherical bubbles armored by birefringent surfactant crystals can be identified. This behavior implies Pickering-type stabilization.^15^Figure S8 presents the normalized volume of foam and drained oil as well as foam temperature *vs* aging time. The fraction of residual foam after 1-month storage for both systems ranges between 85 and 95%, while that of drained oil is <10%. The photos of the foams after 3 months aging are given as insets in the figure. They behave as firm gels. The evolution in appearance of rapeseed oil foams containing 20 wt % Span 60 during storage at 7 °C or at room temperature is displayed in Figure 7. Virtually no change in the appearance of the foams can be identified after 1-month storage, with no oil drainage or foam collapse. In addition, crystal-coated air bubbles can be observed from polarized microscopy images. The average bubble diameter after 1 week and 1-month storage at 7 °C is plotted as a function of surfactant concentration in Figure S9. It decreases gradually and then levels off with increasing surfactant concentration. For 15 and 20 wt % surfactants, the bubble diameter remains more or less constant (20–30 μm) on storage, implying the absence of coarsening/coalescence. Based on the above, we conclude that ultrastable oleofoams of high air volume fraction can be prepared with Span 60. The reasons for exceptional foamability and superior foam stability are as follows. Here, aeration is performed in the one-phase molecular region at high temperatures, in contrast to the majority of previous work reporting the preparation of oil foams through whipping oleogels containing surface-active lipid crystals.^15−28^ The kinetics of adsorption of surfactant monomer to the air–oil surface is faster than that of surfactant crystals, thus facilitating more efficient air incorporation.^43^ Second, the viscosity of oleogels is several orders of magnitude higher than that of oil solutions. A high viscosity tends to limit the amount of air bubbles beaten into continuous oil.^44^ Third, rapid cooling triggers local crystallization of surfactant molecules at bubble surfaces and in the continuous phase.^29^ The former can improve the interfacial elasticity of air bubbles, thereby hindering bubble dissolution and coalescence;^45^ the latter can induce gelation of continuous oil (see Figure 3), which can arrest, in particular, drainage and, through it, coarsening and coalescence.^45−47^ As argued before^29^ and shown here using FTIR, a complex of surfactant molecules and TAG molecules forms *via* hydrogen bonding in the one-phase and at air bubble surfaces in foams. It is likely that some TAG becomes incorporated into surfactant crystals upon cooling the foam, enhancing its stability.

#### Rheology of Stable Foams

The rheology of selected foams containing 10 and 20 wt % Span 60 in rapeseed oil stored at 7 °C was studied by stress sweep and frequency sweep experiments (Figure S10). From the stress sweep measurements, *G*′ is always higher than *G*″ over the investigated stress range and the yield stress (τ_Y_ > 100 Pa) where *G*′ = *G*″ cannot be obtained within the same region. This indicates a firm, solid-like network. It is worth noting that *G*′ of the foam with 20 wt % Span 60 is almost independent of amplitude stress, revealing its exceptional rigidity. Frequency sweep measurements were carried out within the LVR at a fixed oscillation stress of 5 Pa. *G*′ is larger than *G*″ within the studied frequency range. Moreover, *G*′ for the foams with 10 wt % (∼8 × 10^4^ Pa) and 20 wt % Span 60 (∼1 × 10^5^ Pa) is independent of frequency characteristic of a firm gel.

### Whipping Mixtures of Span 80 and Vegetable Oils

**Figure 8:**
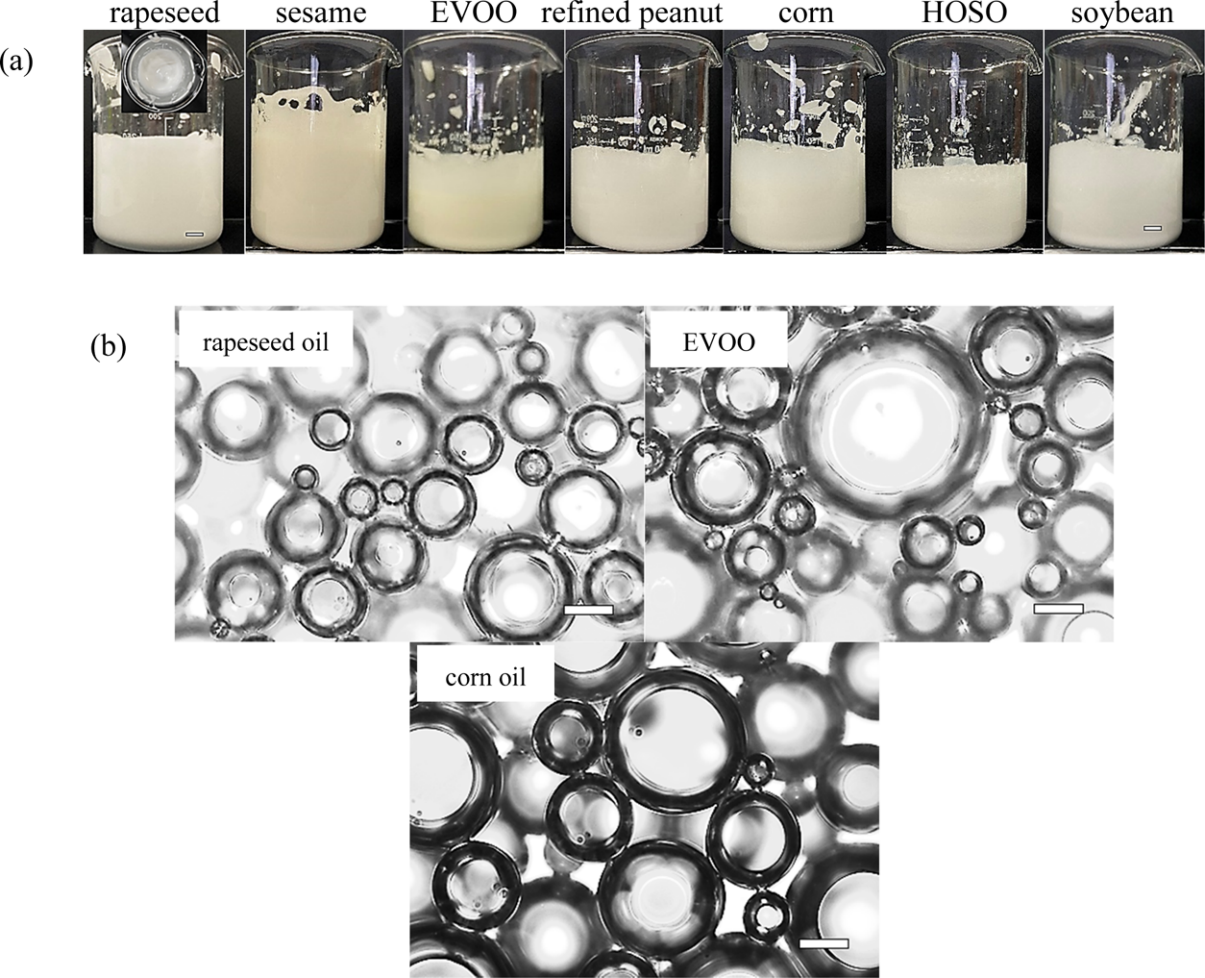
(a) Photos, scale bar = 1 mm and (b) optical micrographs of vegetable oil foams containing 10 wt % Span 80 immediately after 10 min whipping at ambient temperature, scale bar = 50 μm.

To testify whether the above findings are applicable to other sorbitan ester–oil systems, we chose to study the foaming behavior of mixtures of Span 80 and various vegetable oils, including rapeseed oil, sesame oil, extra virgin olive oil (EVOO), refined peanut oil, corn oil, high oleic sunflower oil (HOSO), and soybean oil. The mixtures were first submitted to 10 min whipping at room temperature. Subsequently, the resulting foams were stored at the whipping temperature or cooled rapidly to −5 °C. Figure 8 and Table 1 give the foaming properties of 10 wt % Span 80 in oil during whipping and subsequent storage at room temperature. The highest over-run value is 200% for sesame oil (air volume fraction of 0.67). All foams suffer complete collapse within 7 days, however. In addition, the foam bubbles are spherical and possess smooth surfaces. For rapeseed oil foams stored at −5 °C, they suffer a gradual decay, with only 10–15 vol % of the initial foam surviving after 1 of month aging, Figure S11. From the optical micrograph in Figure S11c, foam bubbles with textured surfaces can be observed. Here, the foam stability is also enhanced upon cooling. Although we have not established the reasons for the different foaming behavior with the different oils, what we can conclude is that the foamability and foam stability both increase with an increase in the saturated fatty acid content within the oil (apart from data for rapeseed oil).

**Table 1:** Foaming Properties of Vegetable Oil Foams Stabilized by 10 wt % Span 80^a^

oil	over-run (%) (±10)	half-life (h) (±2)	time for complete foam collapse (h) (±5)
rapeseed	183	70	168
sesame	200	12	60
EVOO	100	3	30
refined peanut	100	8	72
corn	146	9	48
HOSO	95	3	14
soybean	154	12	60

a Foams prepared at ambient temperature were stored at the same temperature.

For certain surfactant concentrations, the cooled foams made with Span 60 at 7 °C are much more stable than those with Span 80 stored at −5 °C. First, this contrast arises from the difference in the degree of supercooling Δ*T* between the two systems. Δ*T* is defined as the difference between the equilibrium melting temperature of the surfactant (*T*_m_) and the set temperature (*T*_set_).^48^ Δ*T* equals ∼46 °C for Span 60 foams, which is significantly larger than ∼6 °C for Span 80 foams. Larger Δ*T* leads to faster kinetics of surfactant crystal formation on cooling,^48^ thus arresting foam aging more effectively.^49,50^ Second, Span 60 renders the gelation of continuous oil, while Span 80 does not, at the relevant storage temperatures (see Figure S12).

## Conclusions

In this paper, we apply the protocol developed recently to prepare stable edible oil foams of high air volume fraction in mixtures of vegetable oil and sorbitan ester surfactant, demonstrating a novel use of this classical surfactant. Whipping is first performed in the one-phase molecular region, where a complex forms *via* hydrogen bonding between molecules of surfactant and those of TAG, followed by rapid cooling and storing at low temperatures. For high-melting sorbitan monostearate, the resulting foams containing crystal-coated air bubbles are completely stable to drainage, coarsening, or coalescence. In contrast, the cooled foams stabilized by low-melting sorbitan monooleate suffer a gradual decay, but the residual fraction lasts for more than 1 month. This contrast is mainly due to differences in the kinetics of surfactant crystal formation and oil-gelling behavior during cooling. Compared with previously reported oil foams,^15−28^ sorbitan ester-stabilized foams have significantly higher air volume fraction, *i.e.*, lower density, and have potential application in foods and cosmetics. It is anticipated that the mechanism of foam stabilization can be extended to other nonaqueous systems containing hydroxyl-rich surfactant and ester oils.

## Supporting Information Available

Chemical structures of sorbitan esters; DSC curves of neat Span 60; thermal properties of rapeseed oil gels containing Span 60; FTIR spectra of neat Span 60, neat Span 80, rapeseed oil, and rapeseed oil solutions of Span 60; oil foam stability *vs* Span 60 concentration or storage temperature; photographs and polarized microscopy images of Span 60 foams stored at low temperatures; stability of Span 60 foams stored at low temperatures; average bubble diameter of Span 60 foams stored at low temperatures *vs* storage time; rheology of Span 60 foams stored at low temperatures; photographs and optical microscopy images of Span 80 foams stored at low temperatures; and appearance of rapeseed oil solutions of Span 80 stored at low temperatures (PDF)
