# Supplementary material for: Fabrication of Stable Oleofoams with Sorbitan Ester Surfactants
Source: Langmuir. 2022 Nov 21;38(48):14779–88. doi: 10.1021/acs.langmuir.2c02413 (PMC9730906; doi:10.1021/acs.langmuir.2c02413)

Supporting Information

FABRICATION OF STABLE OLEOFOAMS WITH SORBITAN ESTER SURFACTANTS

Yu Liu and Bernard P. Binks*

Department of Chemistry, University of Hull, Hull. HU6 7RX. UK

* b.p.binks@hull.ac.uk

No. Figures 12
No. Tables 2

Figure S1. Chemical structure of (a) sorbitan monostearate (Span 60), (b) sorbitan monooleate (Span 80). Each surfactant molecule has three free hydroxyl groups.

(a)

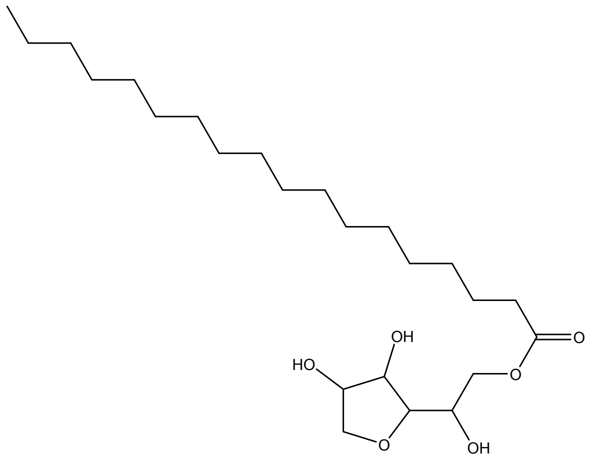

(b)

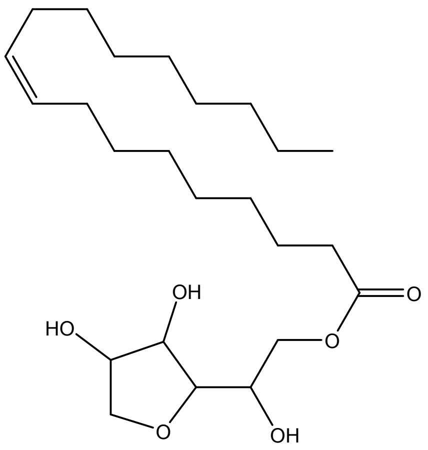

Figure S2. DSC melting (upper trace) and cooling (lower trace) thermograms of neat Span 60. Temperature change rate was 5 °C min^{-1}.

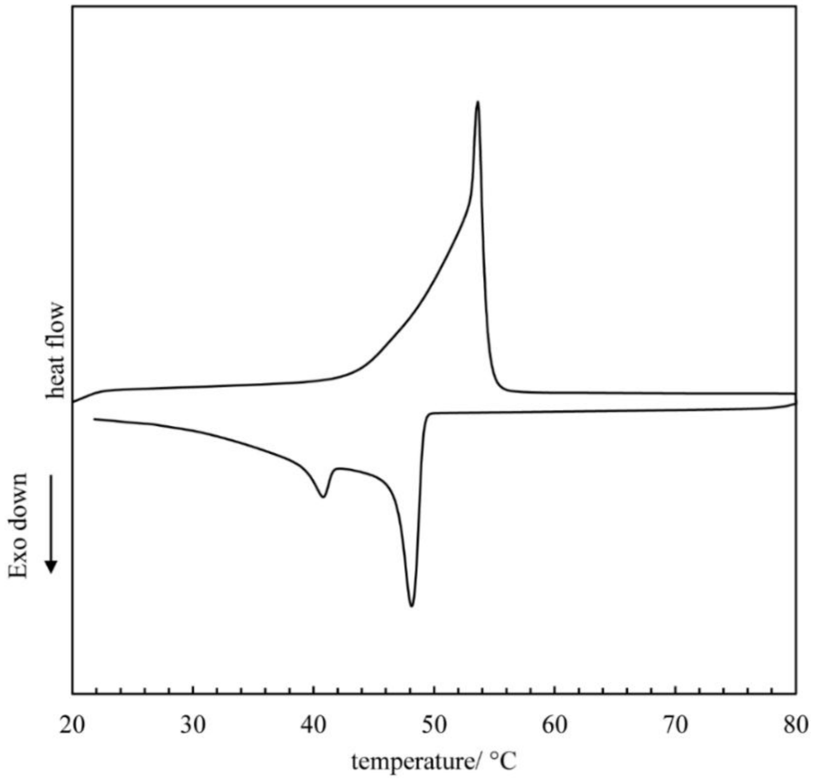

Table S1. Thermal properties of Span 60.

Crystallisation				Melting	
T_{peak}/°C		ΔH/J g^{-1}		T_{peak}/°C	ΔH/J g^{-1}
Peak 1	Peak 2	Peak 1	Peak 2	Peak 1	Peak 1
40.70 ± 0.08	48.20 ± 0.04	-4.93 ± 0.18	-30.31 ± 2.14	53.40 ± 0.20	95.24 ± 2.33

Table S2. Thermal properties of rapeseed oil gels containing different concentrations of Span 60 stored at room temperature.

[Span 60]/wt.%	Crystallisation		Melting	
	T_{peak}/°C	ΔH/J g^{-1}	T_{peak}/°C	ΔH/J g^{-1}
5	No peak	No peak	No peak	No peak
10	45.5 ± 0.1	1.7 ± 0.3	50.1 ± 0.2	2.0 ± 0.5
15	46.0 ± 0.2	4.5 ± 1.7	50.6 ± 0.1	5.1 ± 1.6
25	46.6 ± 0.1	6.7 ± 1.0	51.1 ± 0.2	8.1 ± 2.0

Figure S3. (a) Peak temperature and (b) phase transition enthalpy as a function of Span 60 concentration in rapeseed oil during crystallization (blue circles) and melting (orange circles).

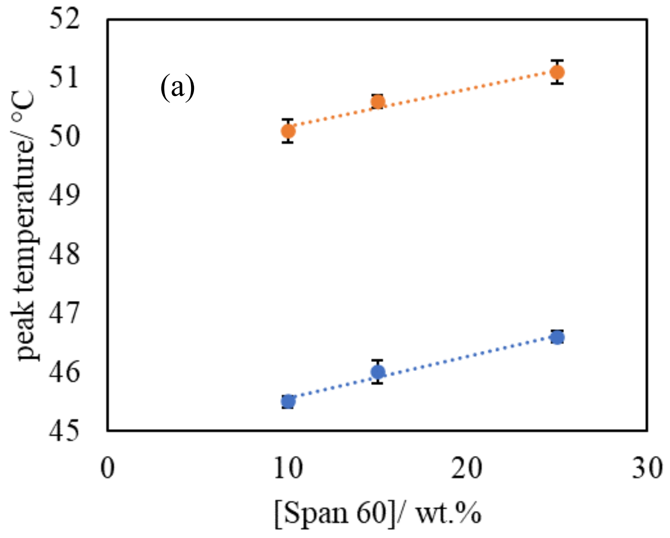

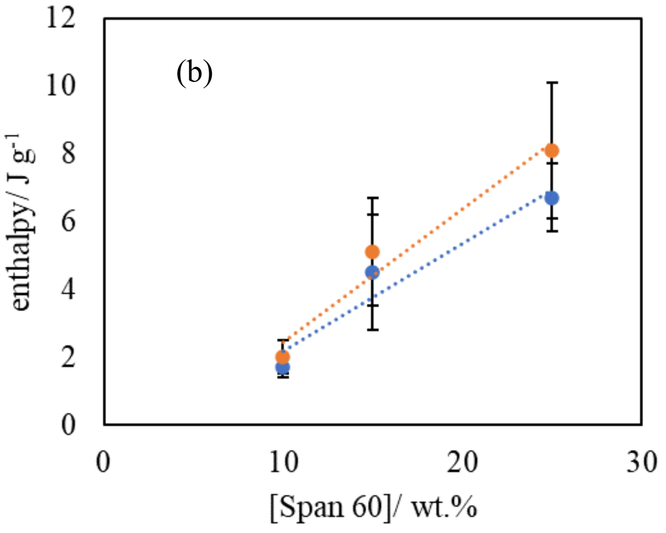

Figure S4. FTIR spectra of (a) neat Span 60 and (b) neat Span 80 and rapeseed oil (RO) at room temperature, (c) variation of absorbance at 3475 cm^{-1} with Span 60 concentration in rapeseed oil for systems in Figure 4(b) at 70 °C (one-phase).

(a)

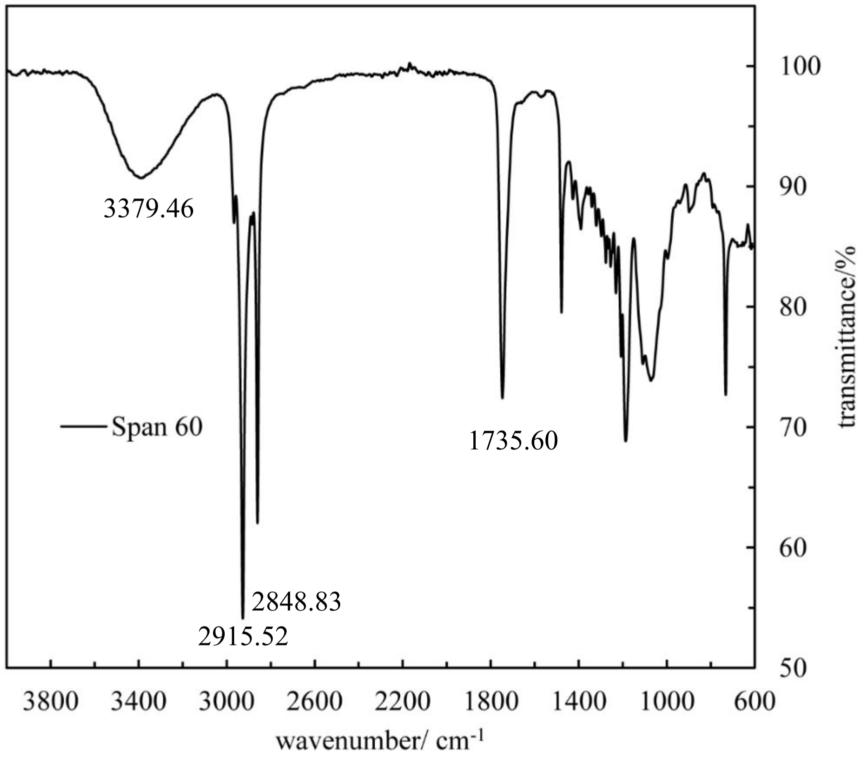

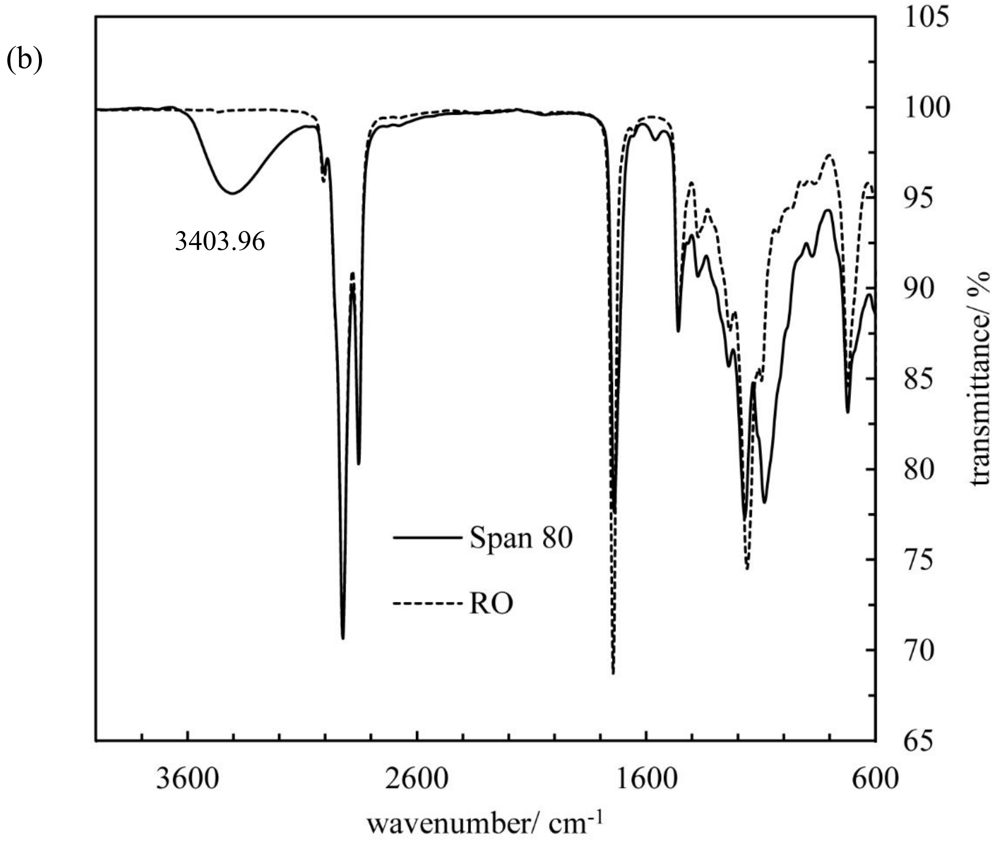
(b)
3403.96
Span 80
RO
wavenumber/ cm-1
transmittance/ %
3600
2600
1600
600
105
100
95
90
85
80
75
70
65

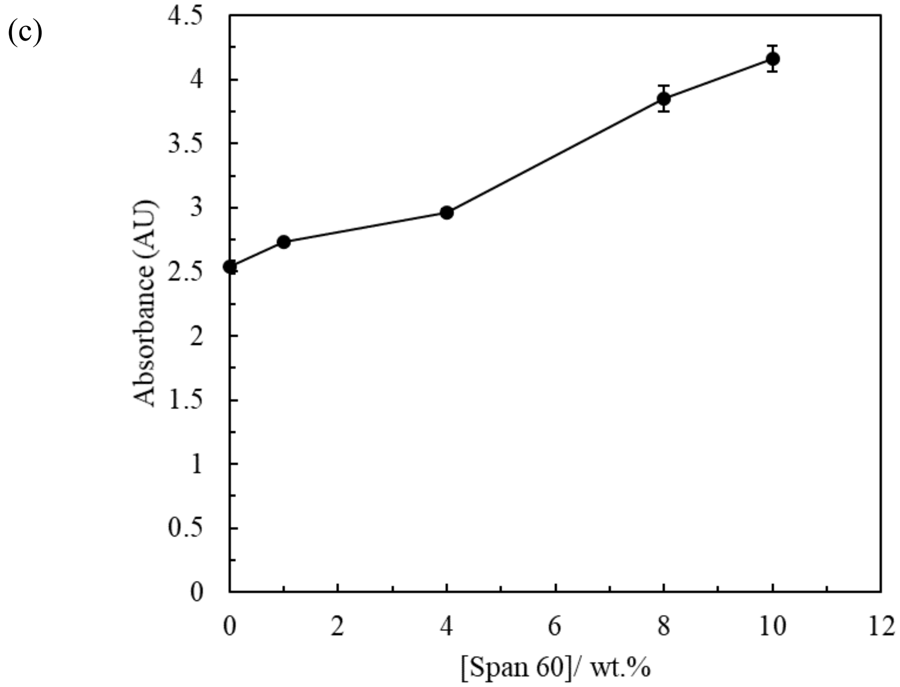
(c)
Absorbance (AU)
[Span 60]/ wt.%
0
0.5
1
1.5
2
2.5
3
3.5
4
4.5
0
2
4
6
8
10
12

Figure S5. Foam half-life (filled points) and time for complete foam collapse (open points) against (a) storage temperature and (b) concentration of Span 60 for systems in Figure 5. Storage temperature is the same as whipping temperature.

(a)

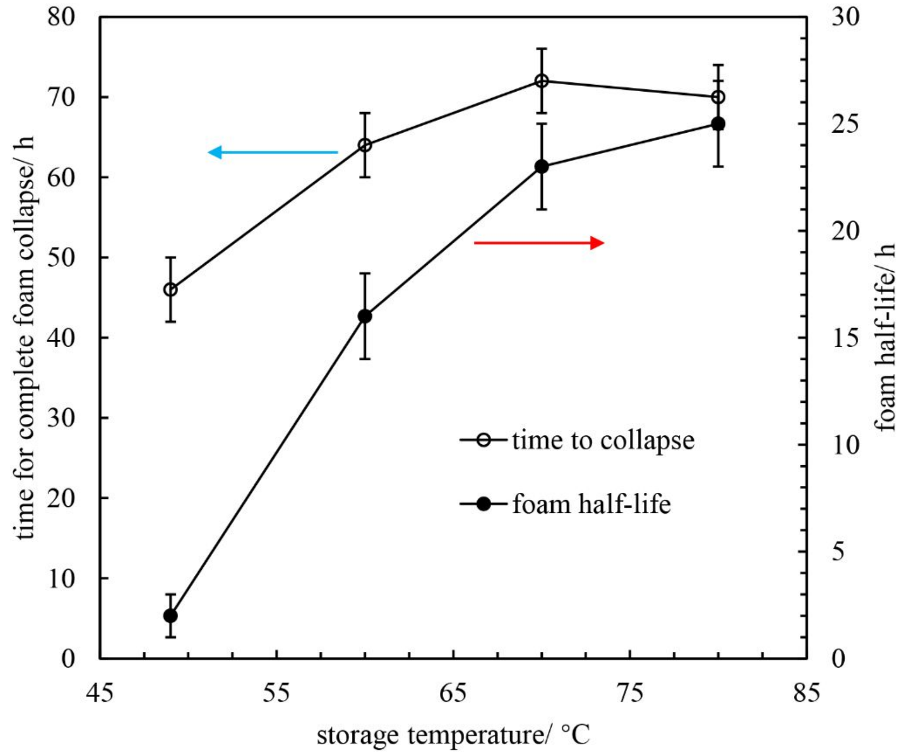

(b)

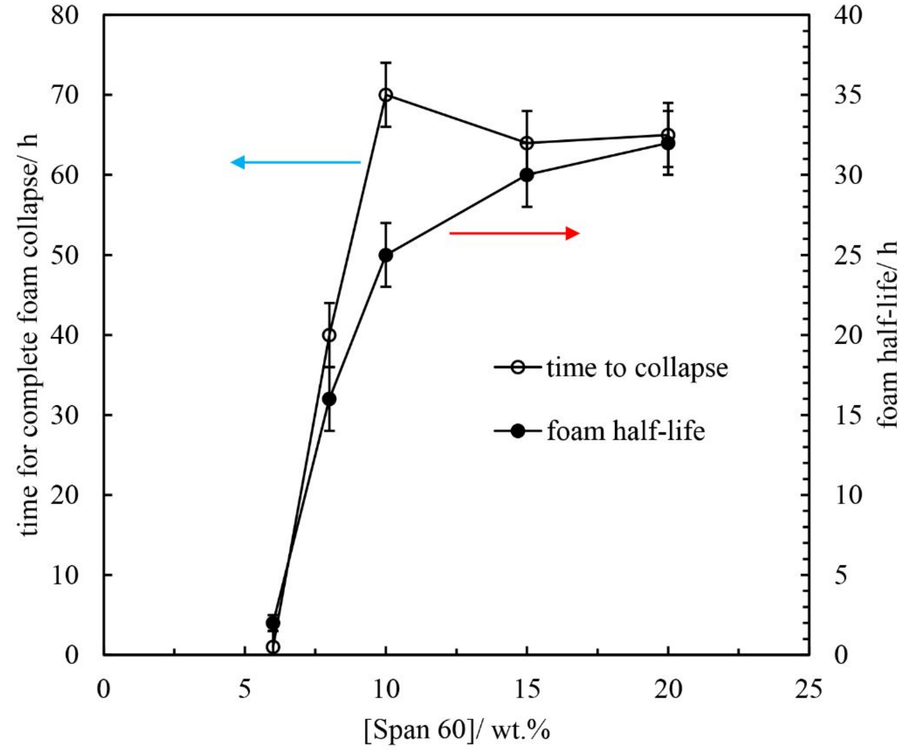

Figure S6. (a) Appearance of rapeseed oil foam containing 10 wt.% Span 60 submitted to rapid cooling immediately after 10 min whipping. Foam prepared at 80 °C was cooled in an ice bath of -5 °C followed by storing at 7 °C. Scale bar = 1 cm. (b) Non-polarized (left) and polarized (right) microscopy images of the foam in (a) after 1 month storage at 7 °C. Scale bars = 50 μm.

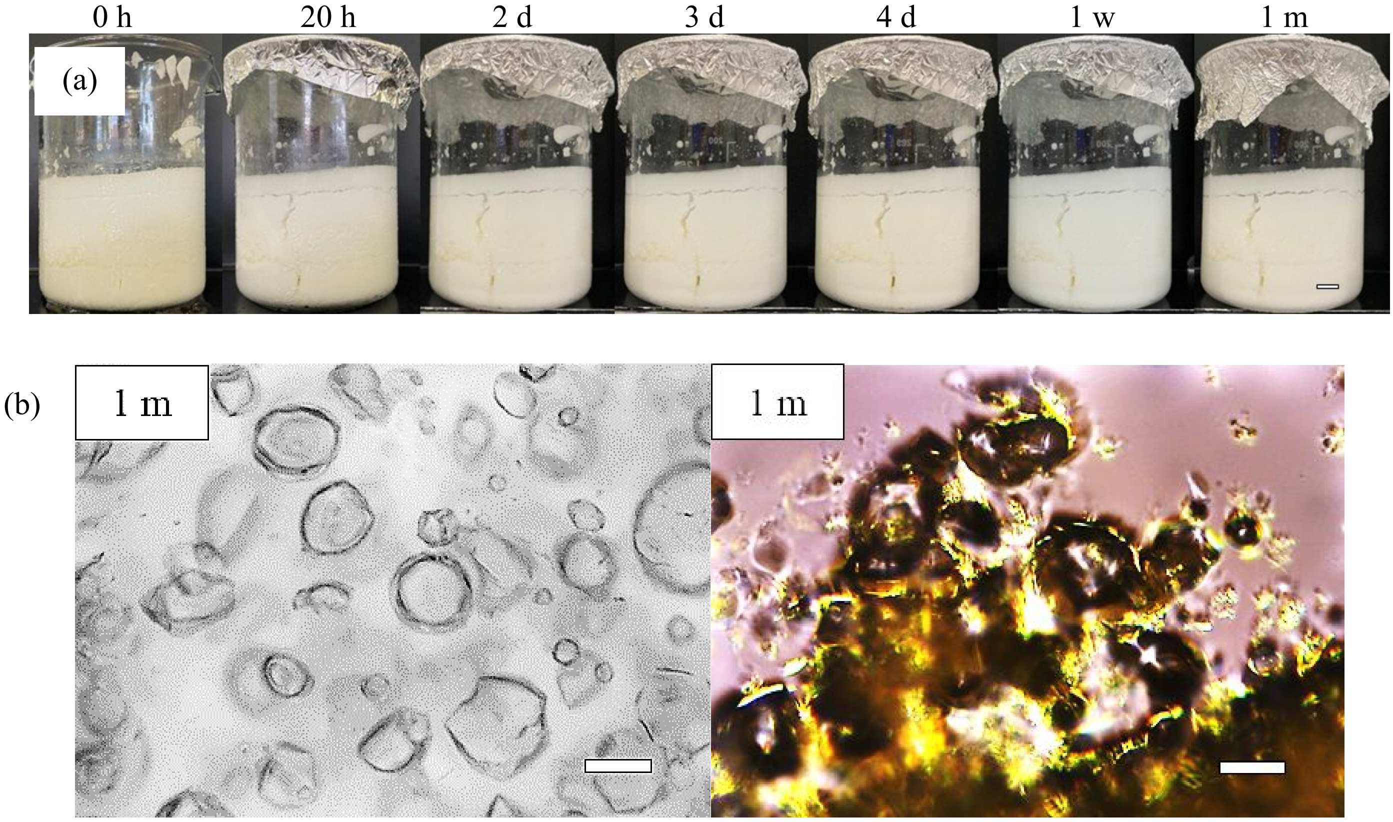

Figure S7. (a) Appearance of rapeseed oil foam containing 15 wt.% Span 60 submitted to rapid cooling immediately after 10 min whipping. Foam prepared at 80 °C was cooled in an ice bath of -5 °C followed by storing at 7 °C. Scale bar = 1 cm. (b) Non-polarized (left) and polarized (right) microscopy images of the foam in (a) after 1 month storage at 7 °C. Scale bars = 50 μm.

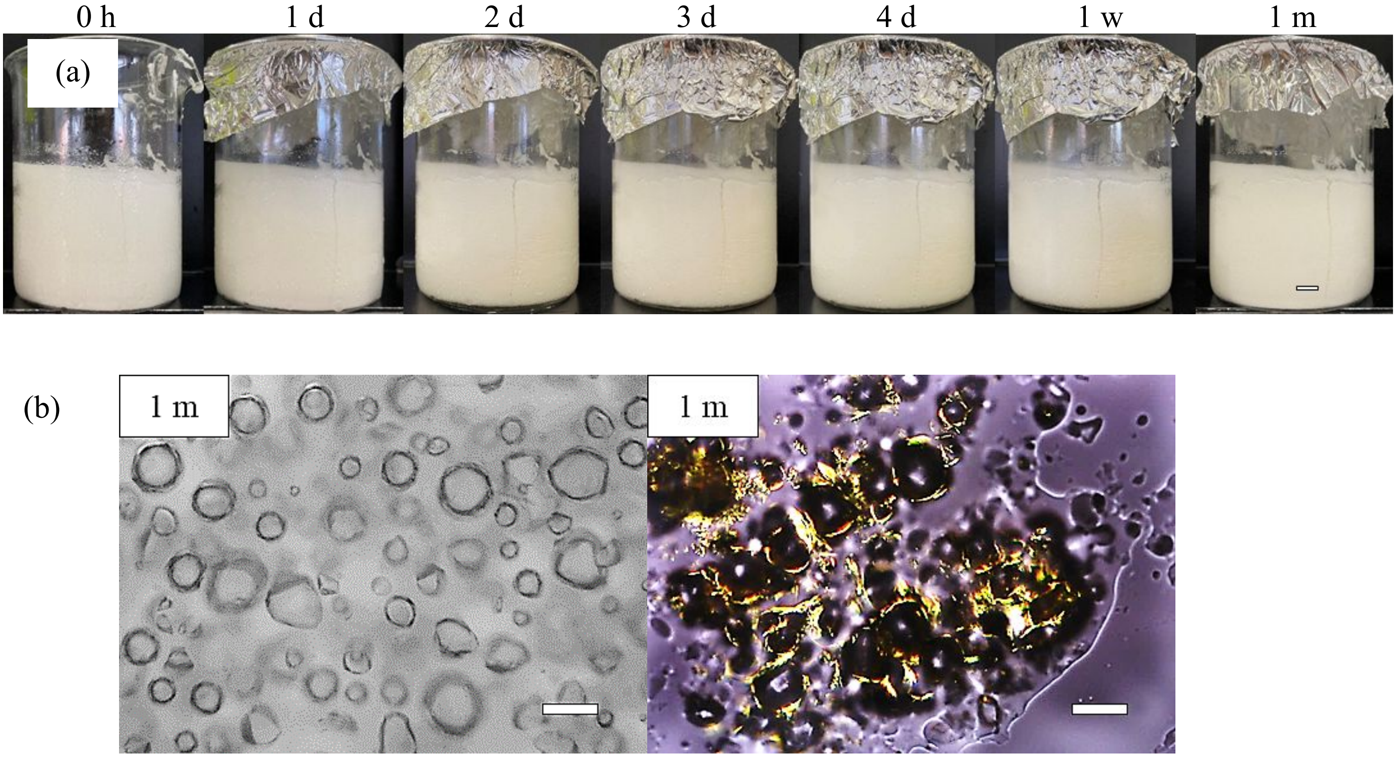

Figure S8. Normalised volume of foam (●) and drained oil (○) and foam temperature (■) as a function of aging time for the systems in Figures S6 and S7. Inset: Photo' of the foam after 3 months storage, scale bar = 1 cm. Foams prepared at 80 °C were cooled in an ice bath of -5 °C followed by storing at 7 °C. (a) 10 wt.% Span 60, (b) 15 wt.% Span 60 in rapeseed oil.

(a)

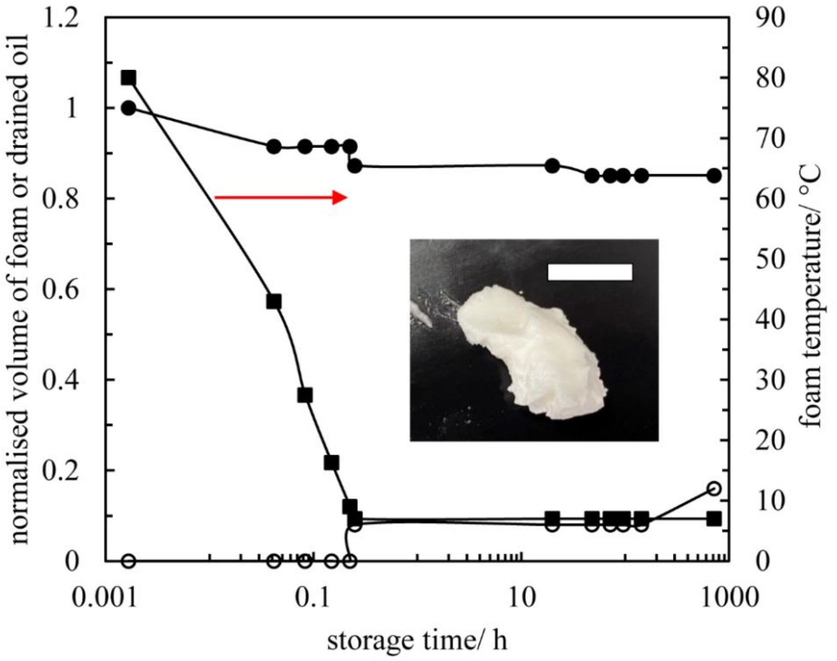

(b)

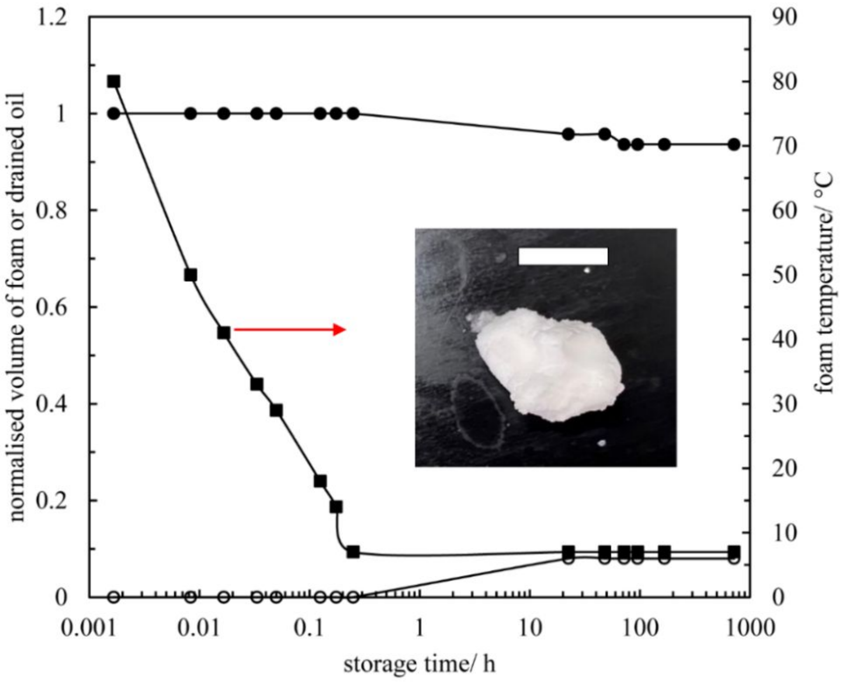

Figure S9. Average bubble diameter of rapeseed oil foams as a function of Span 60 concentration after (○) 1 week and (●) 1 month storage at 7 °C. Foams prepared at 80 °C were cooled in an ice bath of -5 °C followed by storing at 7 °C.

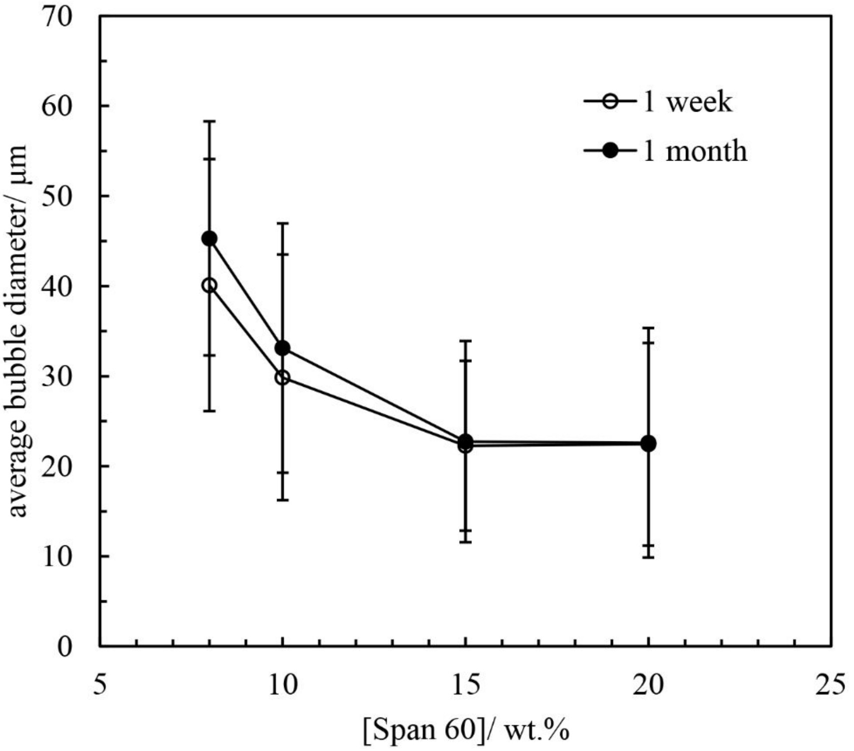

Figure S10. Rheology of rapeseed oil foams containing 10 wt.% (squares) or 20 wt.% (circles) Span 60 after 1 month storage at 7 °C. (a) Amplitude sweep at a fixed frequency of 1 Hz, (b) frequency sweep at fixed stress of 5 Pa within the linear viscoelastic region.

(a)

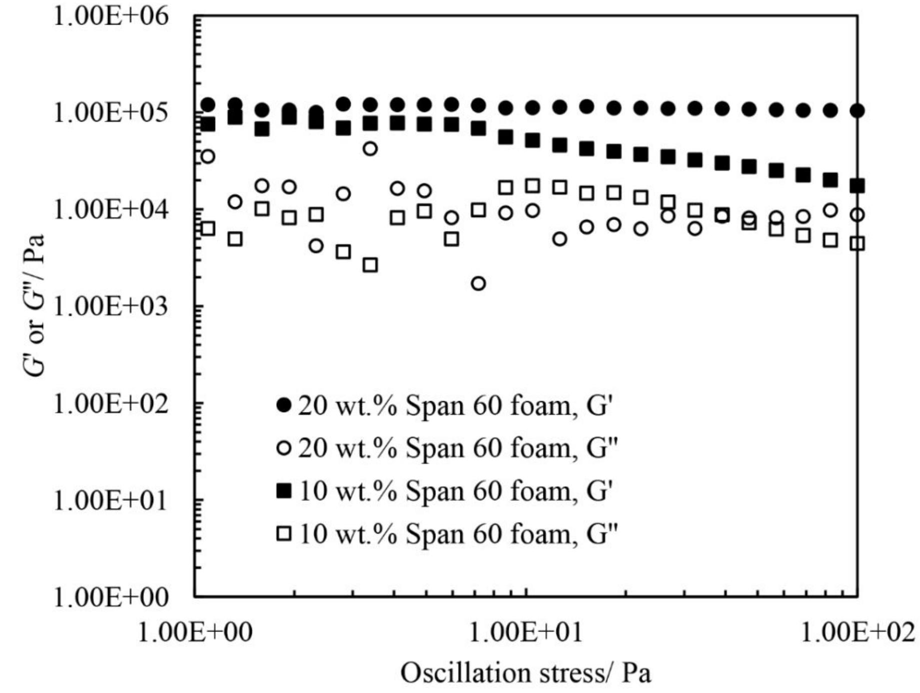

(b)

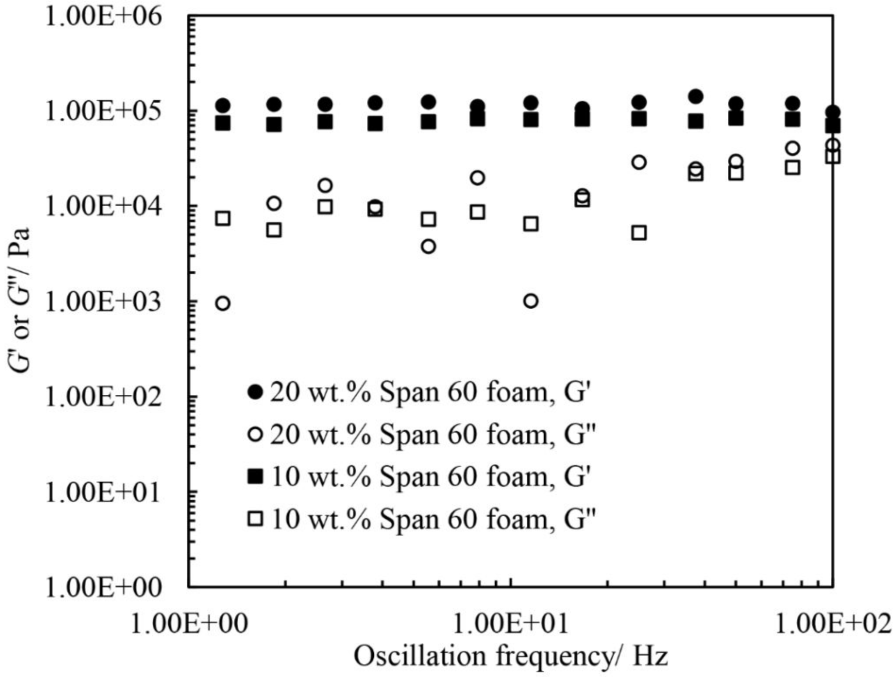

Figure S11. Photos of rapeseed oil foams containing (a) 15 wt.% and (b) 20 wt.% Span 80 submitted to rapid cooling immediately after 10 min whipping. (c) Optical micrograph of the foam in (b). Foams prepared at room temperature were cooled in an ice bath of -5 °C followed by storing at -5 °C. Scale bars equal 1 cm and 50 μm for photos and micrograph, respectively.

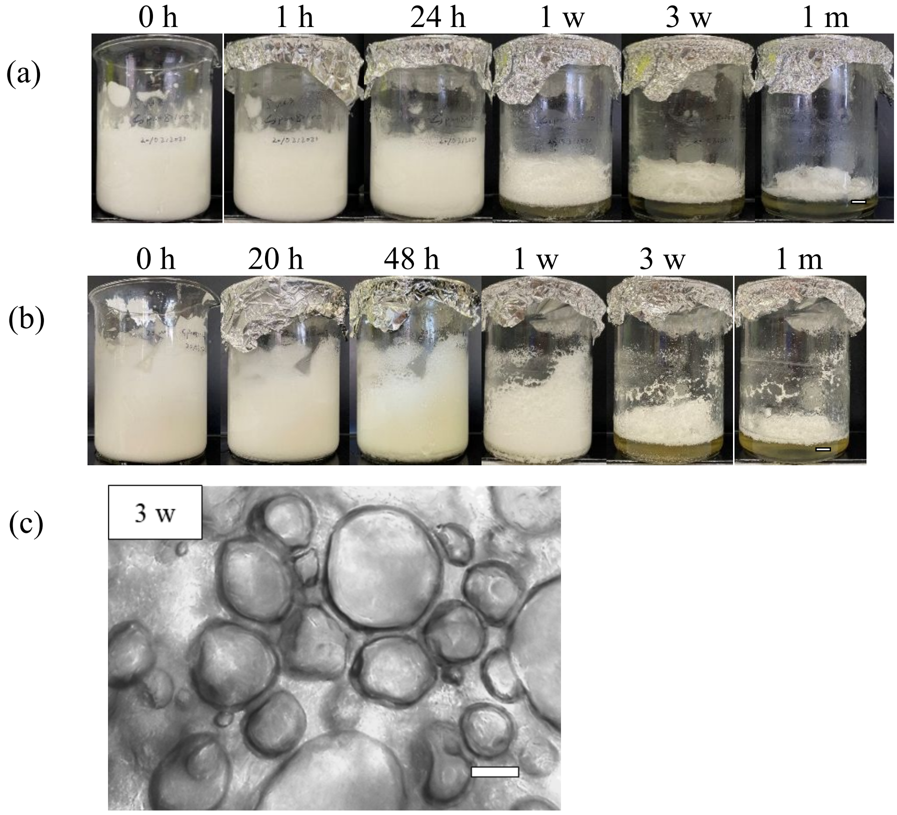

Figure S12. Appearance of vials containing different concentrations of Span 80 in rapeseed oil after 24 h storage at -5 °C cooled from room temperature: (a) 5 wt.%, (b) 10 wt.%, (c) 20 wt.%. Scale bar = 1 cm.

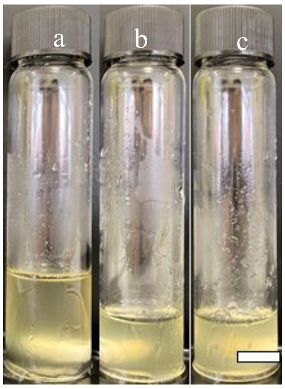

Supplement: Supplementary file 1 — la2c02413_si_001.pdf [file la2c02413_si_001.pdf]
